# Human embryos harbor complex mosaicism with broad presence of aneuploid cells during early development

**DOI:** 10.1038/s41421-024-00719-3

**Published:** 2024-09-24

**Authors:** Fan Zhai, Siming Kong, Shi Song, Qianying Guo, Ling Ding, Jiaqi Zhang, Nan Wang, Ying Kuo, Shuo Guan, Peng Yuan, Liying Yan, Zhiqiang Yan, Jie Qiao

**Affiliations:** 1 Center for Reproductive Medicine, Department of Obstetrics and Gynecology, Peking University Third Hospital, Beijing, China; 2 National Clinical Research Center for Obstetrics and Gynecology, Beijing, China; 3 Key Laboratory of Assisted Reproduction (Peking University), Ministry of Education, Beijing, China; 4 Beijing Key Laboratory of Reproductive Endocrinology and Assisted Reproductive Technology, Beijing, China; 5 Research Units of Comprehensive Diagnosis and Treatment of Oocyte Maturation Arrest, Beijing, China; 6 Peking-Tsinghua Center for Life Sciences, Peking University, Beijing, China; 7 Beijing Advanced Innovation Center for Genomics, Beijing, China; 8 Academy for Advanced Interdisciplinary Studies, Peking University, Beijing, China

**Keywords:** Chromosomes, Developmental biology

## Abstract

Pre-implantation genetic testing for aneuploidy (PGT-A) is used in approximately half of in vitro fertilization cycles. Given the limited understanding of the genetics of human embryos, the current use of PGT-A is based on biologically uncertain assumptions and unvalidated guidelines, leading to the possibility of disposing of embryos with pregnancy potential. We isolated and sequenced all single cells (1133) from in vitro cultured 20 human blastocysts. We found that all blastocysts exhibited mosaicism with mitotic-induced aneuploid cells and showed an ~25% aneuploidy rate per embryo. Moreover, 70% (14/20) of blastocysts contained ‘chromosome-complementary’ cells, suggesting genetic mosaicism is underestimated in routine PGT-A. Additionally, the analysis of 20,945 single cells from day 8–14 embryos (in vitro cultured) and embryonic/fetal organs showed that 97% of the analyzed embryos/organs were mosaic. Over 96% of their aneuploid cells harbored ≤ 2 chromosome errors. Our findings have revealed a high prevalence of mosaicism in human embryos.

## Introduction

Pre-implantation genetic testing for aneuploidy (PGT-A) is widely applied in clinical practice, and approximately half of in vitro fertilization cycles undergo PGT-A in the United States^1^. PGT-A is a genetic screening approach for deselecting affected embryos, improving rates of implantation and live birth^2^. Several PGT-A studies have shown that 2%–90% of embryos were diagnosed as having chromosomal mosaicism^2–5^. These studies mostly determined mosaic embryos via intermediate copy number strategy (20%–80% or 30–70%). However, some studies point out that this strategy can cause false positives of mosaic embryos, and that the reported mosaic embryos were actually uniform aneuploid/euploid embryos^6^. This may result in the misguided disposal of potentially viable embryos and unnecessary termination of pregnancies^7^. Although multiple studies evaluated mosaicism in human embryos via various methods^8–11^, single cell genetic characteristic of the whole embryo is still unclear. Studies of both fetal and placental tissues reported a low incidence of mosaicism during first- and second-trimester pregnancies via cytogenetic chromosome analysis of chorion villus sampling (CVS) or amniocentesis sampling^12,13^. However, such a small proportion of cells obtained from CVS or amniocentesis sampling cannot reveal the genetic status of the whole fetus at different developmental stages. Considering the limited access to multiple tissues from the fetus, the dynamic mosaicism alterations during human fetal development remain largely unknown.

In this study, we performed single-cell sequencing and data analysis to explore, for the first time, human embryo/fetus chromosomal mosaicism throughout multiple consecutive developmental stages spanning from the blastocyst stage to 26 weeks of gestation. For human blastocysts, the genetic status of each cell from the embryo was screened. Unexpectedly, 100% (20/20) of blastocysts were found to be mosaics. Likewise, 96% of in vitro cultured day 8–14 human embryos showed mosaicism. Additionally, embryonic/fetal cerebral, heart, and kidney tissues showed widespread mosaicism during the 5–26 week period. We find that the mosaicism incidence is extremely high throughout human embryo-fetal development, and the results of our study underscore the need to apply more tolerance when deciding the utilization of mosaic embryos in the clinic.

## Results

**Fig. 1:**
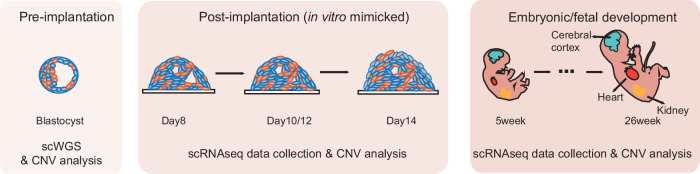
Single-cell sequencing and data analysis to uncover aneuploidy in blastocysts and day 8–14 embryos as well as in embryonic/fetal organs. Schematic illustration of this study. Blastocysts were digested into single cells and single-cell whole–genome sequencing (scWGS) was used to detect CNVs. scRNA-seq data from our previous studies were used to conduct CNV analysis of embryos at day 8–14 stages (post-implantation) and embryonic/fetal organs (including the cerebral cortex, heart, and kidney) spanning 5–26 weeks. Data on pre-implantation, post-implantation, and embryonic/fetal development stages were from different sets of embryos/fetuses.

To comprehensively determine the genetic characteristics of human embryos through development, we performed single-cell sequencing at distinct stages: at the whole-embryo scale in the blastocyst stage, and single-cell RNA-sequencing (scRNA-seq) analysis of embryos at day 8–14 (in vitro cultured) stage and of embryonic/fetal organs spanning 5–26 weeks. The blastocysts were collected from 20 volunteers, with maternal age 30.6 years old on average (range 27–39 years old). Data from post-implantation and embryonic/fetal organs were downloaded from our previous studies^14–17^. All single-cell data were subjected to copy number variation (CNV) detection to determine the aneuploidy pattern of human embryos/fetuses (Fig. 1).

### Sequencing of all single cells from the whole embryos reveals that all in vitro cultured blastocysts are mosaic

**Fig. 2:**
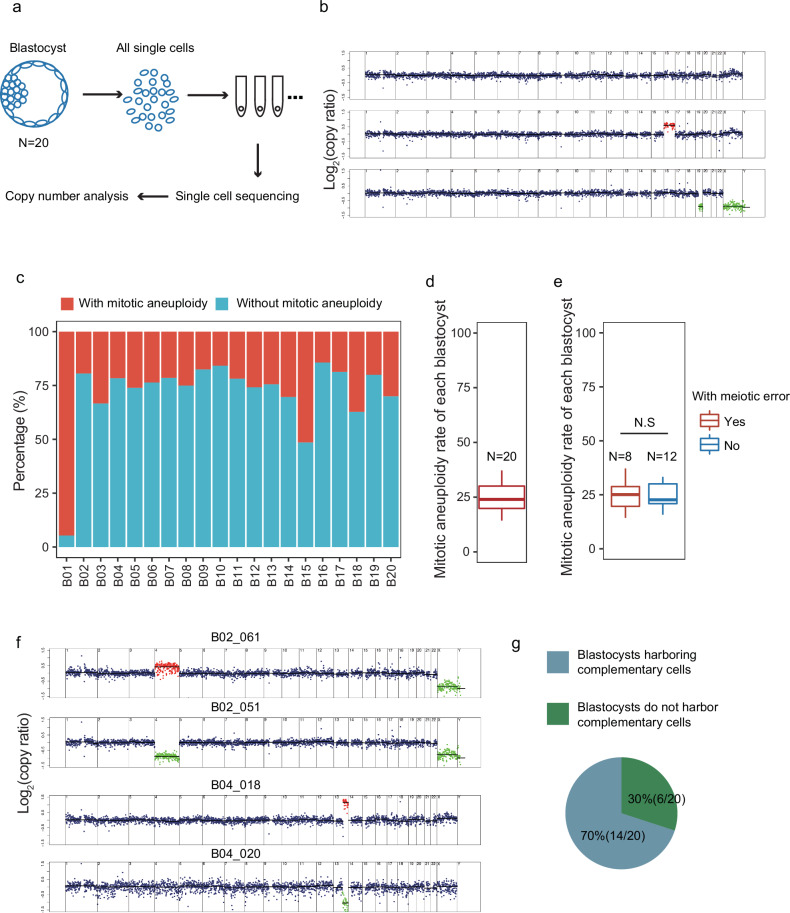
Aneuploidy characteristics at the whole-embryo scale at the blastocyst stage. **a** Approach for detecting aneuploidy in all single cells of blastocysts by scWGS. N number of blastocysts. **b** Representative segmentation plots of euploid (normal number of chromosomes) and aneuploid (chromosome gain and chromosome loss) cells. The top plot is an example of euploid cells, and the middle and bottom plots are examples of chromosome gain (colored in red) and loss (colored in green). **c** Stacked bar plot showing the mitotically induced aneuploidy rate in each blastocyst. **d** Boxplot showing the aneuploidy rate in the blastocyst stage. N number of blastocysts. **e** Boxplot showing the aneuploidy rates of blastocysts with and without meiosis-induced aberrations. N number of blastocysts. N.S. not significant, (*t*-test, *P*-value = 0.76). **f** Segmentation plots showing ‘complementary’ chromosome gain and loss (whole chromosome and segmental chromosome) at the single-cell level. **g** Pie charts showing the percentage of blastocysts harboring chromosome ‘complementary’ cells.

To investigate mosaicism frequency, we collected 20 blastocysts from 20 couples. Each blastocyst was digested, mechanically blown into single cells with a mouth pipette, and then all single cells visible under a microscope were harvested (Fig. 2a; Supplementary Fig. S1a). In total, 1133 blastocyst single cells were collected. Whole-genome sequencing (WGS) at 0.3× depth was then performed on each collected single cell. The variability score (VS) of each cell was calculated to determine the quality of the cells (Supplementary Fig. S1b, c). ~5.38% (61/1133) of sequenced cells exhibited aberrantly high VS (Supplementary Fig. S1b) and were excluded to avoid the possible impact of biased amplification of fragments during WGS. After passing quality control, the CNVs of 1072 cells were determined (Fig. 2b; Supplementary Fig. S1d and Table S1). The accuracy of the single-cell CNV detection approach was validated by screening 14 embryos from chromosome translocation families with known karyotypes, involving segmental and whole chromosome aneuploidies (Supplementary Table S2).

Aneuploidy can result from meiotic or mitotic errors. Meiotic errors, which affect gametes and the resulting zygotes, are expected to yield uniform aneuploidy in all cells of the individual embryo. Mitotic aneuploidies meanwhile affect a fraction of cells, depending upon the timing of their occurrence. Therefore, the aneuploidy pattern determined from analysis of all embryonic single cells in individual embryos can help distinguish between meiotic and mitotic errors^8^. In this study, we scored meiotic-origin aneuploidies as present when ≥ 95% of cells of an embryo displayed a uniform aneuploidy. Among the 20 blastocysts examined in our study, 12 embryos showed only mitotic-origin aneuploidies, 8 embryos showed both assumably meiotic and mitotic aneuploidies, and no embryos showed only assumably meiotic aneuploidies (Supplementary Table S1).

Since mosaicism was mainly due to mitotic error, only mitotic chromosome aberrations were included, and chromosomes exhibiting assumably meiotic errors were excluded from further analysis. For example, in blastocyst B17, chromosome 16 (chr16) showed uniform trisomy, but other chromosomes did not harbor uniform aneuploidies. Therefore, chr16 of blastocyst B17 was excluded from further analysis, while the other chromosomes were retained. CNV analysis revealed that all 20 collected blastocysts were mosaic (harboring cells with different karyotypes) and showed an average 25% mitotic-origin aneuploidy rate per embryo (Fig. 2c, d). The mitotic aneuploidy rate of each embryo showed no significant difference between the embryos with and without assumably meiotic aneuploidy (Fig. 2e). Additionally, the mitotic aneuploidy rate was not correlated with the maternal age in the blastocyst stage (Supplementary Fig. S1f), which is consistent with previous studies^18^. In each blastocyst, 5.36% of cells contained only chromosome gains, 14.29% of cells contained only chromosome losses, and 3.49% of cells contained both chromosome gains and losses (Supplementary Fig. S2a). On the other hand, 9.43% of cells contained only whole chromosome aberrations, 8.71% of cells contained only segmental chromosome aberrations, and 0.47% of cells contained both whole and segmental chromosome aberrations in each blastocyst (Supplementary Fig. S2b). Interestingly, we found that 70% of (14/20) blastocysts contained cells with ‘complementary’ aneuploidy (random chance < 0.001); e.g., in blastocyst B02, B02_061 cell included chr4 gain while B02_051 cell of this embryo included chr4 loss (Fig. 2f, g; Supplementary Table S1). Such ‘complementary’ aneuploidy might be masked in routine PGT-A based on multicell biopsies and bulk DNA assays. These results suggest that the frequency of mosaicism in human blastocysts may have been underestimated in previous PGT-A studies.

During cleavage of the human early embryo, chromosomal instability is relatively common. The topologies of the phylogenies can reveal the pattern of cell divisions during embryo development. Having obtained the majority of cells from the blastocysts, we were able to reconstruct a comprehensive phylogeny of each blastocyst. For instance, in B14, we determined the karyotype of all 33 single cells. Identical and complementary chromosome aberrations were used to construct the phylogeny of development. We inferred that the asymmetric cell division of B14 occurred at ~8-cell to morula stage. Twenty-three single cells of B14 showed the normal karyotype (46, XX), while 5 cells showed chr14 aneuploidy, 3 cells showed chr7 aneuploidy, and 2 cells showed chrX aneuploidy (Supplementary Fig. S3). We inferred that chr14 aneuploidy occurred at ~8-cell stage. One mother cell at the 8-cell stage produced daughter cells with chr14 loss and chr14 gain. In addition, daughter cells with chr14 loss survived with the retained karyotype, while daughter cells with chr14 gain showed other novel aberrations.

### Single-cell analyses show widespread mosaicism of embryos at days 8–14 (in vitro cultured embryos)

Given that aneuploid elimination in mammalian embryo development has provoked intense debate^19–22^, we further focused on analyzing embryonic mosaicism at the subsequent developmental stage. Recent studies have demonstrated that scRNA-seq data can be used to generate accurate digital chromosome karyotypes. To identify chromosome aberrations in day 8–14 embryos, we used high-quality scRNA-seq data that we previously generated from the analysis of the same developmental period of human embryos^14^. Using this data, we employed a strategy similar to the one described by Starostik et al., which was based on allelic imbalance and gene expression alterations^8^. To identify a more reliable threshold to determine chromosome aneuploidy, we included the scRNA-seq data from 280 embryonic cells produced by a combined single-cell genome-and-transcriptome strategy. To ensure the accuracy of the reported aneuploidy by scRNA-seq, only whole chromosome aneuploidy inferred from scRNA-seq data was included. We set stringent criteria to define chromosome aberrations (details in Matherials and Methods). In this way, 27 chromosome aneuploidy events were identified by scRNA-seq data and the 25 of them were also confirmed by corresponding scDNA-inferred CNVs, lending confidence to the aneuploid events reported by this method (Supplementary Fig. S4).

**Fig. 3:**
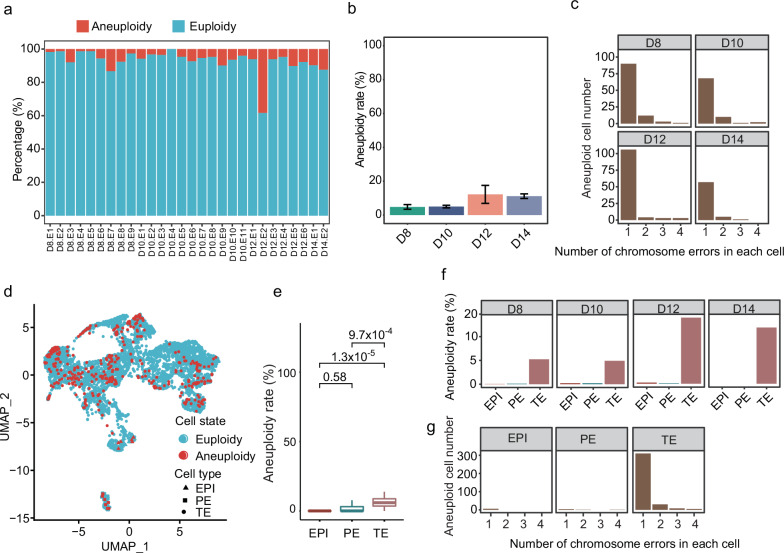
Aneuploidy characteristics in embryos at the day 8–14 stage. **a** Stacked bar plot showing the aneuploidy rate of each embryo on day 8 (D8), day 10 (D10), day 12 (D12), and day 14 (D14). **b** Bar plot showing the aneuploidy rate on D8, D10, D12, and D14. The error bars refer to standard deviation (SD). **c** Bar plot showing the distribution of aneuploid cells with different numbers of chromosome errors on D8, D10, D12, and D14. **d** Uniform manifold approximation and projection (UMAP) showing the aneuploidy distribution in EPI, PE, and TE cells. **e** Box plot showing the aneuploidy rate of EPI, PE, and TE cells. The *P*-value was calculated by Kruskal–Wallis test. **f** Aneuploid cell number in EPI, PE, and TE cells on D8, D10, D12, and D14. **g** Bar plot showing the distribution of aneuploid cells with different numbers of chromosome errors in EPI, PE, and TE cells.

Using the verified method described above, we analyzed the scRNA-seq data of 4820 cells from 28 embryos at the day 8–14 stage obtained in our previous study^14^. We inferred the aneuploidy of each single cell and found that 27/28 (96.43%) embryos harbored aneuploid cells (Fig. 3a). The average aneuploidy rates on days 8, 10, 12, and 14 embryos were 4.81%, 5.05%, 12.23%, and 11.15%, respectively (Fig. 3b). As increased chromosome aberrations would raise the genetic burden of cells, we counted the number of chromosome aberrations in each cell. Over 87.70% of aneuploid cells harbored only one chromosome aberration (Fig. 3c), suggesting the possible negative selection of cells with multiple chromosome aberrations. To investigate the aneuploid cell distribution among different cell types, the cells were clustered into epiblast (EPI), primitive endoderm (PE), and trophectoderm (TE) cells. EPI, PE, and TE cells showed 2.51%, 4.96%, and 7.97% aneuploidy rates, respectively (Fig. 3d–f). TE showed significantly higher aneuploidy rate than EPI and PE, while the aneuploidy rate in EPI was comparable with that in PE, indicating the highest aneuploidy tolerance in TE during embryo development (Fig. 3e). Additionally, the aneuploid cells in the EPI had only one chromosome aberration in each cell (Fig. 3g). Compared to PE and TE cells, EPI cells showed the lowest aneuploidy rate, which may be due to negative selection of aneuploid cells in EPI.

### Single-cell analyses reveal mosaicism in nearly all embryos/fetuses spanning 5–26 weeks

**Fig. 4:**
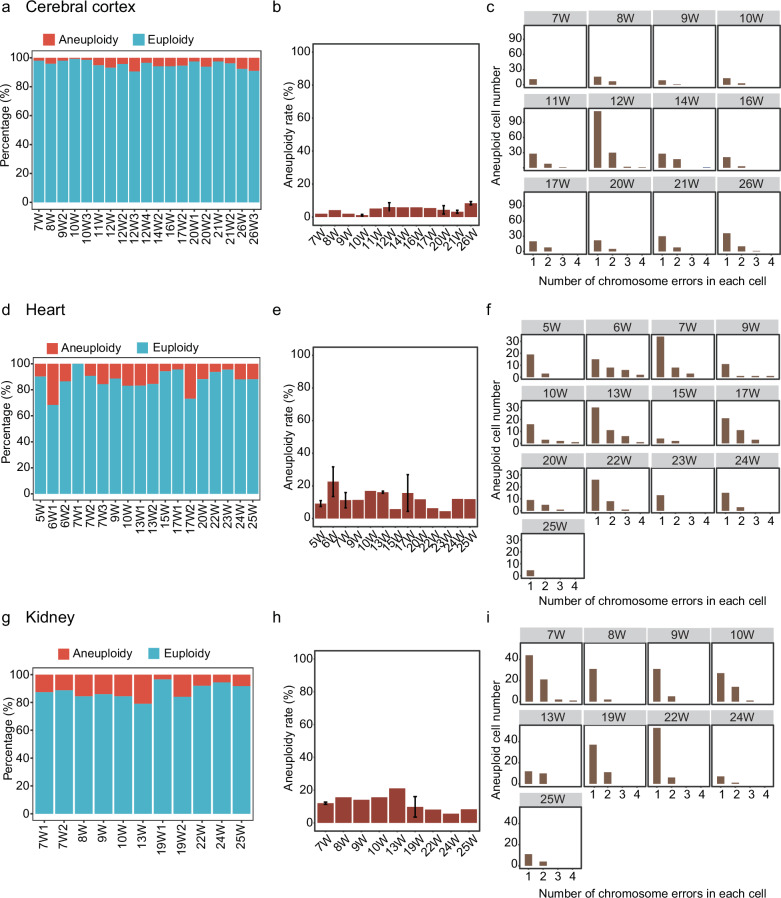
Aneuploidy characteristics in embryonic/fetal organs during development. **a** Stacked bar plot showing the aneuploidy rate in the cerebral cortex of each embryo/fetus spanning 7–26 weeks. **b** Bar plot showing the aneuploidy rate of the embryonic/fetal cerebral cortex in each week. The error bars indicate the SD of aneuploidy rate in each week. **c** Bar plot showing the distribution of aneuploid cells with different numbers of chromosome errors in the embryonic/fetal cerebral cortex. **d** Stacked bar plot showing the aneuploidy rate in the heart of each embryo/fetus spanning 5–25 weeks. **e** Bar plot showing the aneuploidy rate of embryonic/fetal hearts in each week. The error bars indicate the SD of aneuploidy rate in each week. **f** Bar plot showing the distribution of aneuploid cells with different numbers of chromosome errors in embryonic/fetal hearts. **g** Stacked bar plot showing the aneuploidy rate in the kidney of each embryo/fetus spanning 7–25 weeks. **h** Bar plot showing the aneuploidy rate of embryonic/fetal kidneys in each week. The error bars indicate the SD of aneuploidy rate in each week. **i** Bar plot showing the distribution of aneuploid cells with different numbers of chromosome errors in embryonic/fetal kidneys.

To explore the mosaic characteristics of fetal development, we performed CNV analysis on 48 human embryos/fetuses spanning 5–26 weeks at single-cell resolution. The data of these embryos/fetuses were obtained from our previous studies^15–17^. Single-cell data including 10,308 cells from the cerebral cortex of 19 embryos/fetuses, 2990 cells from 18 hearts of embryos/fetuses, and 2827 cells from the kidneys of 11 embryos/fetuses were analyzed. By leveraging the signatures of expression alteration and allelic imbalance, we identified the ploidy status of each cell. We found that 97.92% (47/48) of embryos/fetuses were mosaic (Fig. 4). Embryonic/fetal cerebral cortex cells maintained a low aneuploidy level of less than 10% at each developmental stage (4.70% on average) (Fig. 4a, b). Among cerebral cortex aneuploid cells, only 1.33% harbored > 2 anomalies (Fig. 4c).

Analogous to the cerebral cortex, embryonic/fetal hearts maintained an aneuploidy rate of 12.46% on average (Fig. 4d, e), whereas the fetal kidney showed an 11.98% aneuploidy rate on average (Fig. 4g, h). Compared to cerebral cortex cells, fetal heart and kidney cells showed significantly higher aneuploidy rates, which indicated kidney and heart can tolerate more chromosomal aberrations per cell (Fig. 4f, i; Supplementary Fig. S5).

### Retrospective sequencing of backup biopsy cells reveals mosaicism in PGT-A euploid blastocysts that developed into live births

**Fig. 5:**
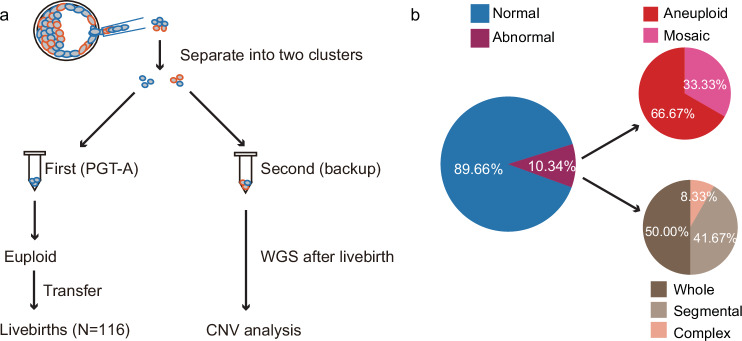
Mosaicism in the backup biopsy samples from embryos with euploid PGT-A and healthy live births. **a** Schematic illustration showing aneuploidy detection in backup biopsy samples. **b** Pie chart showing the abnormal rate (left), aneuploidy rate (top right), and aneuploidy type (bottom right) in the backup biopsy samples.

The collected TE biopsy cells during the PGT-A procedure in our center are routinely separated into two clusters (the first for PGT-A, the second for backup). We collected another 116 PGT-A backup biopsy samples for sequencing and CNV analysis (Fig. 5a). These samples were obtained from blastocysts that were already developed into healthy live births. The CNV analysis results from these backup biopsy samples showed that 10.34% (12/116) contained aneuploid cells. Among them, 8 samples showed aneuploidy of all cells, and 4 samples showed mosaicism (mosaic levels were 44%, 42%, 42%, and 44%, respectively). In 12 samples harboring aneuploid cells, 6 samples showed whole chromosome aberrations (Fig. 5b), suggesting that mosaic embryos with whole chromosome aberrations may have an equal developmental potential compared with embryos with segmental chromosome abnormalities. This retrospective analysis first uncovered the nonnegligible mosaicism associated with healthy live births that were missed at their pre-implantation stage.

## Discussion

Previous studies reported that the usage of PGT-A does not improve the cumulative live birth rate^23^. Although embryos with uniform meiotic aneuploidy are selected away, mosaicism may be one of the most important factors that influence live birth rates with the use of PGT-A. The routine PGT-A is only based on a biopsy of a few TE cells. PGT-A reported high-level mosaic or aneuploidy embryos were disposed of in the clinic, these embryos may show euploid cells in ICM and have the potential to develop into live birth. Multiple methods have been applied to assess human embryo mosaicism in the last two decades^24–27^. Most previous studies were based upon multicell biopsies and reported a mosaicism incidence in pre-implantation embryos ranging from 2% to 90%. Differences in analysis platforms and the limited number of cell samples overshadowed the knowledge of human embryo mosaicism. In addition, many PGT-A programs no longer report mosaicism and do not consider this in embryo selection. Recent studies have shown tolerance to aneuploidy in human early embryos^19,22^. The mosaicism characteristics of human embryos remain elusive and the utilization of mosaic embryos in clinical practice is still controversial. Therefore, understanding the characteristics of embryo mosaicism is essential to provide useful guidance on the transfer or disposal of these embryos in the clinic.

In this study, all single cells from each blastocyst were isolated and sequenced. Our study showed that 100% (20/20) of blastocysts were mosaic, substantially higher than almost all previous PGT-A studies. A recent study utilized haplotyping analysis to identify aneuploidy and the meiotic/mitotic origin of chromosome aberrations, reporting a low level of mosaicism in embryos^2^. This may mainly be due to the different sample collection compared with our study. In a recent study, Starostik et al.^8^ used scRNA-seq data to analyze 74 morula and blastocysts, finding that 59 of the 74 (80%) embryos harbored at least one aneuploid cell, even when analyzed with partial embryonic cells of individual blastocysts. Therefore, the actual mosaicism incidence of blastocysts would be higher when including all single cells of the embryo. In addition, among the 20 blastocysts in this study, 70% (14/20) contained chromosome ‘complementary’ cells. This implied that euploid embryos reported in previous PGT-A studies might be a mixture of euploid and aneuploid cells containing ‘complementary’ chromosome gains and losses. Therefore, the real mosaicism may be masked in the PGT-A assays using multicell biopsies, suggesting the probable underestimation of mosaicism incidences in previous PGT-A studies. Moreover, a PGT-A study on only a small portion of biopsy cells of the embryo cannot represent the whole chromosome constitution, resulting in the undercounts of aneuploid cells of the embryo.

It has been postulated that the remaining euploid cells in the mosaic embryo support the embryo to live birth^28,29^. However, there is currently no direct evidence to support this assumption, and the underlying mechanism during this process remains unclear. Our study provides evidence that mosaicism caused by chromosome copy number alteration is also prevalent in day 8–14 human embryos. Since some mosaic embryos can develop into healthy live births, we speculate that the mosaicism of some chromosomes may not affect development due to the tolerance mechanism of the individual. Thus, we further explored the dynamics of mosaicism during days 8–14 (in vitro cultured) and fetal organ development stages. Previous studies provided valuable resources for us to understand embryonic/fetal development^15–17,30^. Our study estimated the aneuploidy rate of post-implantation embryos and embryonic/fetal organs based on 20,945 single cells spanning day 8–26 weeks of development from previous studies. The day 8–14 embryos showed widespread aneuploidy, indicating that mosaicism exists throughout the post-implantation development. Our results showed a significantly higher mosaicism incidence in embryonic/fetal organs compared to the reported ~1%–2% mosaicism incidence of fetuses in prenatal tests via CVS^31^. This might be mainly due to limited tissues or cells obtained from fetuses in previous studies. Cao et al. utilized single-cell analysis to examine various fetal tissues during the second trimester, which provided a valuable dataset for understanding early human embryonic development^30^. We found that the prevalence of aneuploidy in the embryonic/fetal brain was less than 10%. McConnell et al. reported that 13%–41% of neurons harbored CNVs in the adult frontal cortex^32^, which was higher than our result from embryonic/fetal organs. Likewise, Kristin et al.^33^ and Andrew et al.^34^ reported that 5%–50% of hepatocytes were aneuploid. This suggested human organs can tolerate aneuploid cells both in fetal and adult organs.

Based on these findings, we speculate that aneuploid cells may exist from the pre-implantation stage to live birth. We used backup biopsy cells to retrospectively detect the ploidy status of healthy live births at the blastocyst stage. We found that 10.34% (12/116) of embryos were mosaic, although they were diagnosed as euploid in the PGT-A phase, suggesting that several healthy live births from ‘euploid’ transfer were actually from ‘mosaic’ transfer. This leads us to conclude that the developmental potential of mosaic embryos is greater than previously thought. Multiple studies reported healthy live birth after mosaic embryo transfer, few abnormal phenotypes were observed^35–37^. Combining these results, we suggest that the mosaic embryo should be more acceptable in the PGT-A procedure, especially for those couples that do not have uniform euploid embryos for transfer.

There are also some limitations. In this study, the copy numbers of embryos in the post-implantation stage and fetal organs were inferred based on scRNA-seq data, which may yield false positives and false negatives of the detected CNVs in the study. We set stringent criteria to determine aneuploid cells by scRNA-seq data, which may result in an underestimation of the aneuploidy rate. In addition, the mosaicism incidence rates of post-implantation embryos and fetuses were inferred from partial cells of individuals, which may mask the real incidence reflected by our results.

The disposal of mosaic embryos largely affects the improvement of the live birth rate in the clinic. Our study demonstrates that mosaicism is a natural feature of human embryos and exists throughout human early development. A subset of mosaic blastocysts has the potential to develop normally into live births. Therefore, the use of mosaic embryos should be more acceptable in the practice of assisted reproductive technology for couples without uniform euploid embryos to transfer.

## Materials and methods

### Study design

To investigate the mosaic characteristics of pre-implantation embryo development, 20 blastocysts were collected from 20 couples. Each blastocyst was digested into single cells. Isolated cells were then subjected to WGS, and CNVs were identified. To detect the aneuploidy status along with development, we analyzed four published scRNA-seq datasets including 28 day 8–14 human embryos (9 at day 8, 11 at day 10, 6 at day 12, 2 at day 14)^14^ and 48 embryos/fetuses spanning 5–26 weeks from previous studies^15–17^. The embryonic/fetal organ spanning 5–26 weeks were all from donated embryos/fetuses which were aborted because of the out-plan pregnancy. For day 8–14 stage, cells were randomly picked from digested embryos. For 5–26 week stage, multiple portions of each organ were randomly biopsied and the cells were randomly picked from different sites. Majority of cell types of each organ were included in each dataset, which presented good representativeness for the sampled organs. Single-cell CNV analysis based on RNA-seq data was conducted as previously described^8^.

### Ethical approval

The study was approved by the Peking University Third Hospital Reproductive Ethics Board (2019SZ-025). The donors were fully informed about the purpose of the research. The donors donated the blastocysts on the condition that the donors have other high-quality embryos to transfer or already transferred other embryos in their assisted reproductive technique cycles. All donors signed the appropriate informed consent form. Additionally, the data of post-implantation embryos and fetuses were from our previous publications.

### Single-cell dissociation of human blastocysts

The zone pellucida of blastocysts was removed in PBS with 0.3% HCl for ~2 s and then immediately washed with PBS three times. The nude blastocysts were then transferred into a mixture of 0.25% trypsin-EDTA (Life Technologies) and Accutase (Life Technologies) (1:1) and incubated at 37 °C for 40 min to loosen the cell-to-cell connection of blastocysts. With a glass needle and mouth pipette, the digested clumps were blown into single cells or smaller cell clumps by mechanical friction. For single cells, PCR tubes were used to collect individual cells. The smaller cell clumps were mechanically dissociated into single cells by thinner glass needles. With this method, all single blastocyst cells were collected.

### scWGS

Whole-genome DNA of single cells from blastocysts was amplified and the DNA libraries were constructed using the Universal Library Preparation Kit (Yikon Genomics Inc., China) as we previously described^38^. The constructed DNA libraries were then subjected to next-generation sequencing on the HiseqX platform. Each cell were sequenced in 0.3× depth on average.

### scWGS data processing and CNV analysis

The WGS data obtained from each isolated single cell was processed as previously described^39^. Briefly, the amplification primers, sequencing adapters, and low-quality bases were trimmed by in-house scripts. The trimmed reads were mapped to the human reference genome (UCSC, hg38) using BWA with default settings. The duplicates were removed with Samtools rmdup command and only uniquely mapped reads (MAPQ > 10) were retained for further analysis. The depth and coverage of each cell were evaluated and the cells with < 0.1× depth were excluded.

The chromosome copy number of each single cell was estimated according to a previously reported method with minor modifications. Briefly, the mapped reads were counted within a 1 Mb window using readCounter software. Then, HMMcopy was used to estimate the CNVs by controlling for reference mappability and GC content^40^. The HMMcopy output corrected read counts were further used to evaluate the variability of each cell. The SD of the corrected read counts was computed for each chromosome. The median SD of the 23 chromosomes was defined as the VS for the sample. In this study, cells with low amplification quality, which showed a high data variability, were excluded from further analysis. In detail, the VS of each single cell was calculated and cells with VS > 3 SD (0.34) were discarded. In this way, only cells with high data quality were included in our analysis.

Next, we determined the most reliable cutoffs for chromosome loss (copies = 1), diploidy (copies = 2), and gain (copies = 3). As shown in Supplementary Fig. S1e, the inferred copy number of all chromosomes showed three peaks in the density plot, which correspond to the loss, diploidy, and gain of chromosomes. We calculated the corresponding best partition cutoff of the three peaks, and the cutoff was used to determine loss (log_2_(copy ratio) < –0.45) and gain (log_2_(copy ratio) > 0.35).

In CNV determination, a relatively larger bin can effectively reduce the noise, therefore, we counted the reads in a 1 Mb bin and only CNV larger than 10 Mb were reported. In this way, whole and segmental chromosome error can be reported, which guaranteed the accuracy of the reported CNV. In addition, we found several complementary CNVs in two cells from one embryo, which also confirmed the accuracy of the reported CNV in this study.

### scRNA-seq data processing

The datasets of day 8–14 embryos and embryonic/fetal organs were from our previous studies^14–17^. Theses embryos were obtained at the time of termination. The data were processed as we previously described. Briefly, the template switch oligo (TSO) and poly(A) tail sequences were trimmed, and the sequencing adapters and low-quality bases were further removed using Trim galore (v0.6.5). The processed reads were mapped to the human reference genome (UCSC, hg38) using STAR in two-pass mode. Only uniquely mapped reads (MAPQ > 10) were retained for analysis. Reads on each gene were counted using FeatureCounts (v1.5.3). Genes with counts higher than 1 were defined as expressed genes. To determine cell lineage in the post-implantation stage, Seurat v3.2.3 was utilized to analyze the expression data. Specifically, genes expressed in at least three cells and cells whose expressed genes above 2000 were retained. Expression data were normalized by a scale factor of 100,000. Highly variable genes were determined by the “FindVariableGenes” function, which was used to perform principal component analysis. Twelve PCs were selected as the significant components to perform UMAP using the RunUMAP function. With a ‘resolution’ of 0.6 upon running ‘FindClusters’ function, we distinguished cell types in the UMAP plot according to known markers and identified EPI, PE, and TE cells.

### Aneuploidy detection with scRNA-seq data

The bam files from STAR were further used to call variants and the raw expression matrix was used for further analysis. The CNV of each cell was inferred through the method described by Starostik et al.^8^. Genes expressed in at least three cells and cells whose expressed genes over 2000 were retained for further study. Besides, only genes with median expression > 50 were included for further CNV inference. Additionally, only chromosomes with > 100 SNP split reads were used for CNV inference.

To test the reliability of aneuploidy detection, the scRNA-seq data from 280 embryonic cells produced using a combined single-cell genome-and-transcriptome (scTrio-seq) strategy were analyzed. To ensure the accuracy of reported aneuploidy by scRNA-seq, only whole chromosome aneuploidy inferred from scRNA data was included, which cannot be affected by the reads in low complexity regions. Stringent criteria were set to define chromosome aberrations using the following thresholds: 1) more than 100 SNP split reads were required in each chromosome, 2) *z*-scored gene expression > 5.6 and *z*-scored allelic imbalance > 0.05, identified as chromosome gain, 3) *z*-scored gene expression < –3.65 and *z*-scored allelic imbalance < –1.6, identified as chromosome loss. The accuracy of this approach was assessed using the 280 cells, of which DNA data were used as a golden standard to evaluate the accuracy of aneuploidy inferred from scRNA-seq data. As shown in Supplementary Fig. S4, among the 280 cells, 20 cells harbored chromosome gains or losses via DNA data analysis. In the 20 cells, 388 of 440 autosomes showed high RNA data quality (52 autosomes were excluded for less than 100 SNP reads). Among them, 27 chromosome aberrations were detected in RNA data, of which 25 were confirmed in DNA data, showing a 7.40% false positive rate. Additionally, 361 chromosomes showed no aberrations in RNA data, and 353 of them were confirmed in DNA data, which showed 2.22.% false negative rate.

### PGT-A biopsy backup sample collection and analysis

The PGT-A biopsy cell clumps in our center were routinely separated into two clumps by mouth pipette. The first was used for PGT-A, and the second was deposited as a backup. We retrospectively re-sequenced and analyzed 116 backup samples from blastocysts, which had been diagnosed by PGT-A as euploid based on the first clumps and were then transferred to eventually develop into healthy live births.

## Supplementary information

Supplementary materials

Table S1

Table S2

## Data Availability

All data and protocols in this manuscript are available upon request from the lead contact. The raw sequencing data were deposited in the GSA-human database (HRA003384). The code used in this study is available on github (https://github.com/yzqheart/scBlastocystSeq).
