# Supplementary material for: Human embryos harbor complex mosaicism with broad presence of aneuploid cells during early development
Source: Cell Discov. 2024 Sep 24;10:98. doi: 10.1038/s41421-024-00719-3 (PMC11420220; doi:10.1038/s41421-024-00719-3)
Supplement: Supplementary file 1 — Supplementary materials [file 41421_2024_719_MOESM1_ESM.pdf]

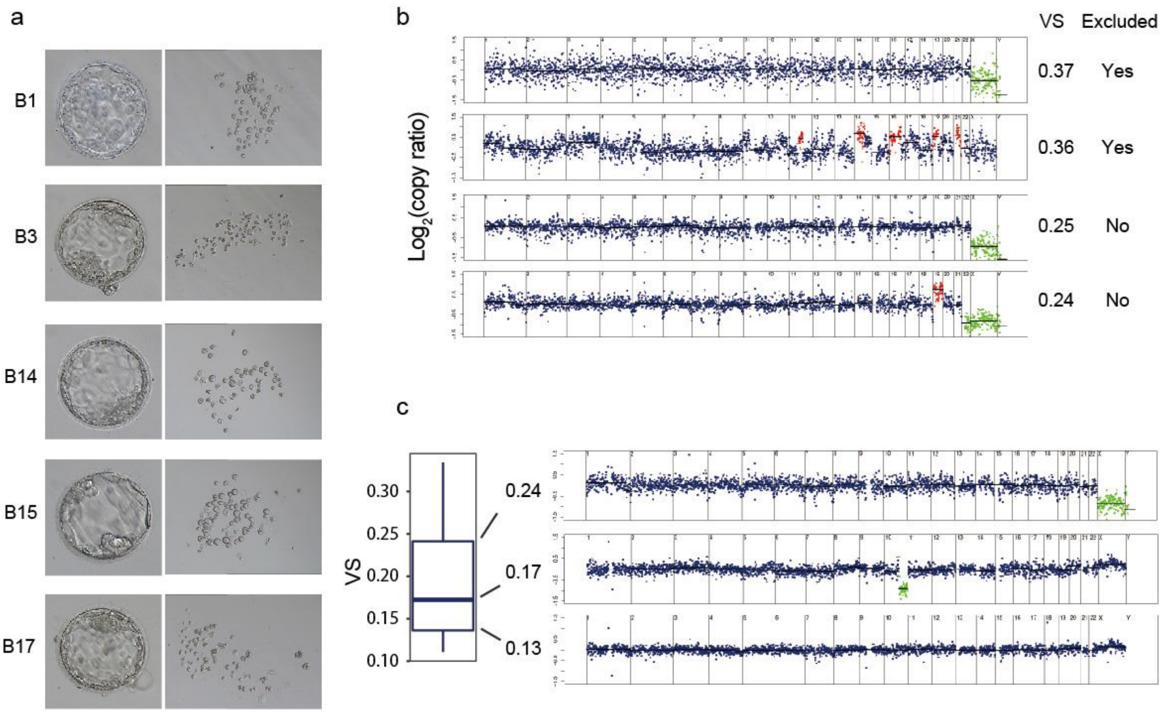

d

	B01	B02	B03	B04	B05	B06	B07	B08	B09	B10	B11	B12	B13	B14	B15	B16	B17	B18	B19	B20
Maternal age	28	29	32	31	32	32	27	NA	28	39	34	39	32	34	38	38	28	31	27	33
Morphological score	4BB	5BC	5BB	4BC	4BC	5BA	4BB	4BB	4BC	4BB	4BA	4BB	5AA	4BC	4BB	4BC	5AB	5AB	4CC	4BC
Harvested	60	70	74	63	74	110	57	39	45	42	64	95	42	33	36	45	77	43	31	33
Analyzed	56	67	72	51	73	106	56	36	40	38	55	93	41	33	35	42	75	43	30	30

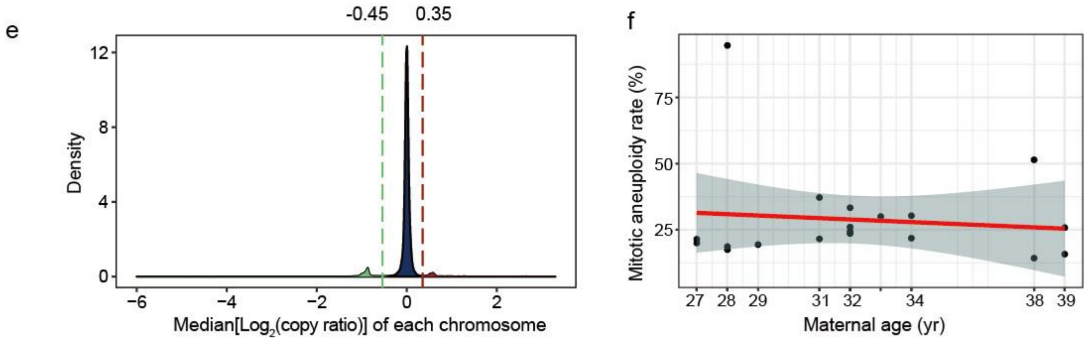

Supplementary figure S1. Single-cell isolation and quality analysis of sequenced cells.

a Microscope images of representative blastocysts and single cells.

b Representative segmentation plots of two cells that were excluded from the analysis (top and upper-middle) and two cells that were included in the analysis (lower middle and bottom). Excluded cells had a wide spread of read counts, which showed high variability scores (VS).

c Box plots of VS for analyzed cells (n = 1,072) (left panel), and representative segmentation plots of three cells with the 25th, 50th, and 75th percentiles of the VS (right panel).

d Table summarizing the corresponding maternal age of each blastocyst and the number of cells that were analyzed in this study. NA, not available.

e Density plot showing the inferred copy number of all chromosomes and the cutoffs for chromosome loss, euploidy, and gain.

f The correlation between maternal age and mitotic aneuploidy rate at blastocyst stage.

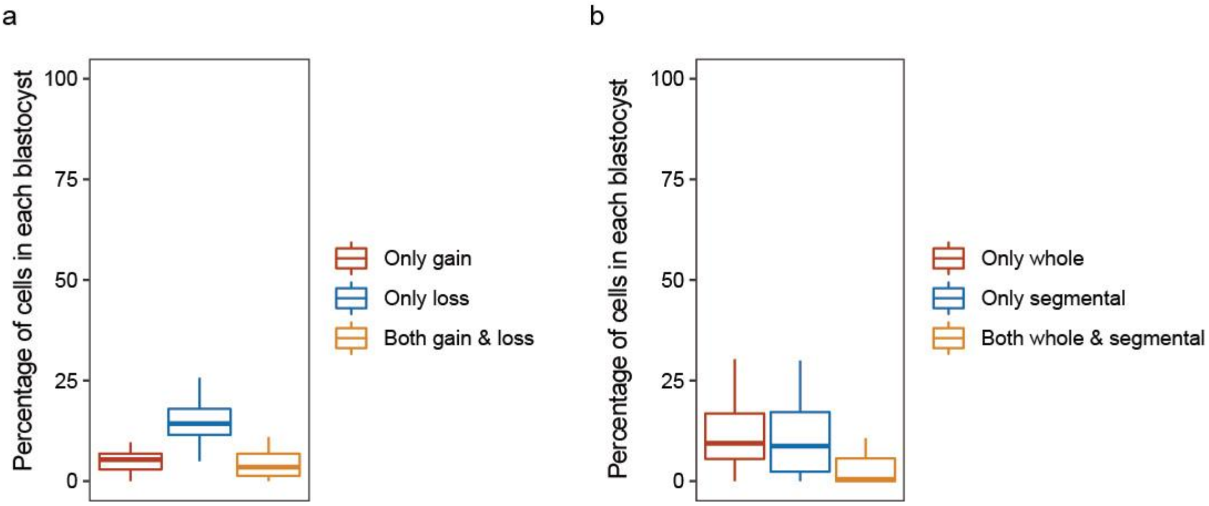

Supplementary figure S2. Chromosome aneuploidy types in blastocysts.

a Box plot showing the percentage of cells containing only chromosome gain, only chromosome loss, and both gain and loss in each blastocyst.

b Box plot showing the percentage of cells containing only whole-chromosome error, only segmental-chromosome error, and both whole and segmental chromosome errors.

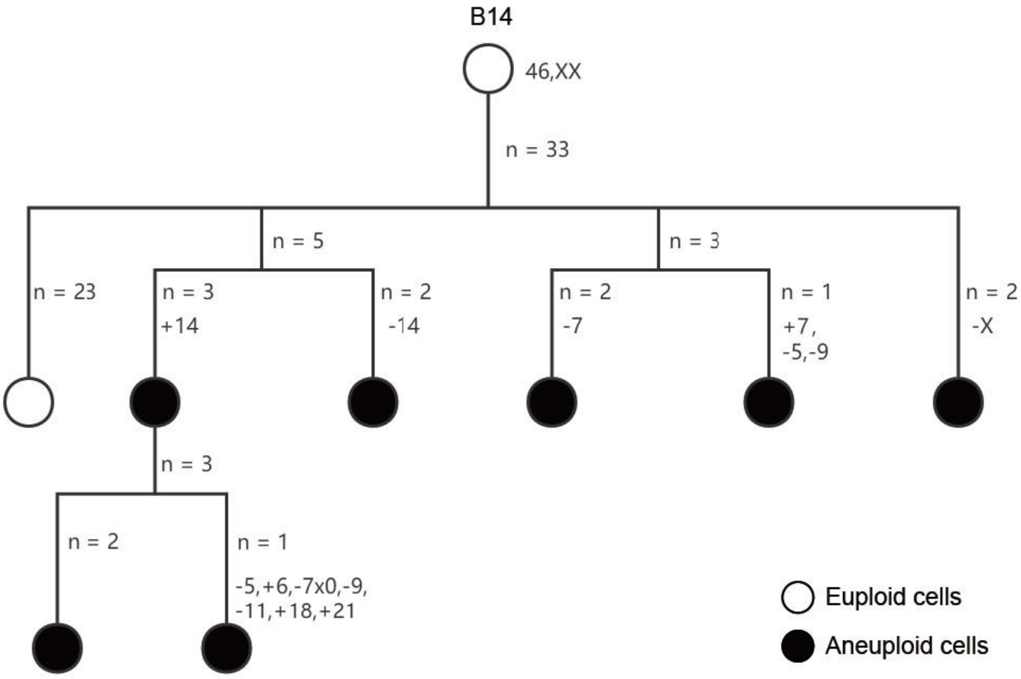

Supplementary figure S3. Hypothetical lineage tree of blastocyst B14.

Lineage tree of B14 as inferred from the karyotype of each single cell.

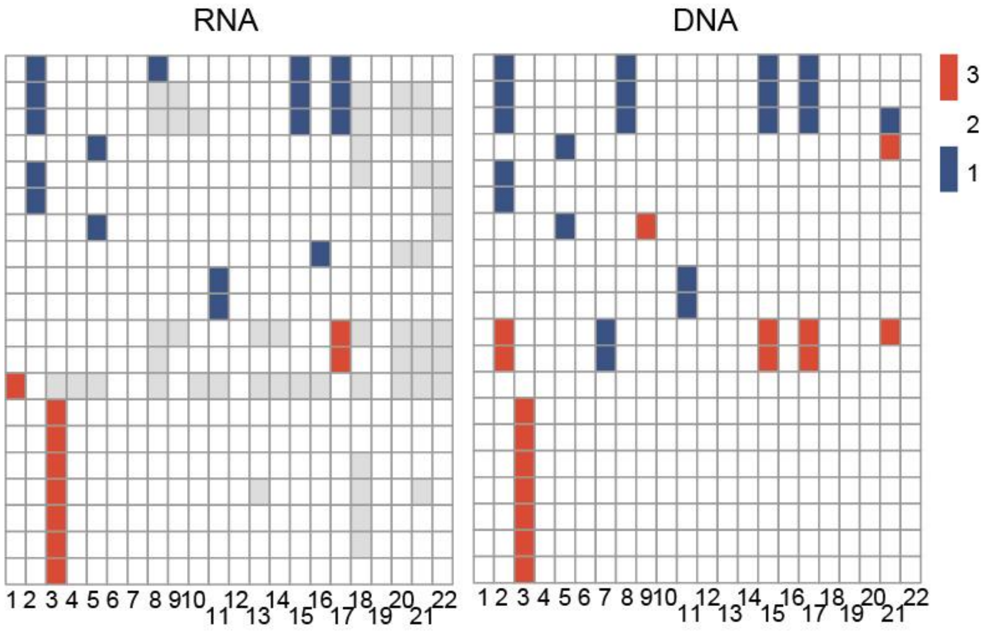

Supplementary figure S4. Concordance between scRNA-seq-inferred aneuploidy and corresponding scDNA-seq-detected aneuploidy.

Heatmap showing inferred aneuploidy from scRNA-seq (left) and corresponding scDNAseq-detected aneuploidy (right). Red represents chromosome gain, blue represents chromosome loss, white represents chromosome euploidy, and gray represents chromosomes with <100 SNP split reads.

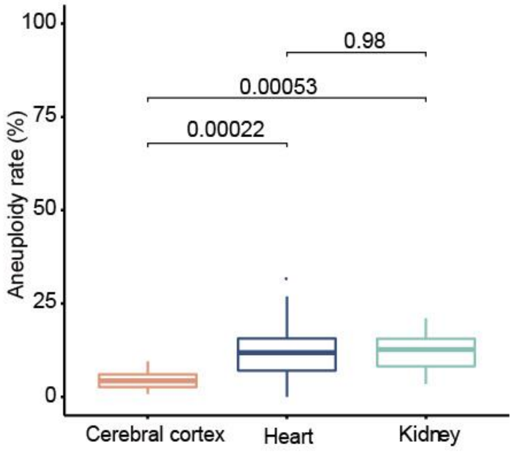

Supplementary figure S5 The box plot showing the aneuploidy rate in cerebral cortex, heart and kidney. P-value was calculated by Kruskai-Wallis test.

Supplementary Table S1. Karyotype of each single blastocyst cell.

Supplementary Table S2. Validation data of known meiotic aneuploidies detected by scWGS.
